# PRL2 Controls Phagocyte Bactericidal Activity by Sensing and Regulating ROS

**DOI:** 10.3389/fimmu.2018.02609

**Published:** 2018-11-13

**Authors:** Cennan Yin, Chenyun Wu, Xinyue Du, Yan Fang, Juebiao Pu, Jianhua Wu, Lili Tang, Wei Zhao, Yongqiang Weng, Xiaokui Guo, Guangjie Chen, Zhaojun Wang

**Affiliations:** ^1^Department of Immunology and Microbiology, Shanghai Jiaotong University School of Medicine, Shanghai, China; ^2^Department of Basic Medicine, Guangxi Medical University, Nanning, China; ^3^Department of General Surgery, Huadong Hospital, Shanghai Medical College, Fudan University, Shanghai, China

**Keywords:** PRL2, oxidative burst, bactericidal activity, Rac GTPase, neutrophil, macrophage

## Abstract

Although it is well-recognized that inflammation enhances leukocyte bactericidal activity, the underlying mechanisms are not clear. Here we report that PRL2 is sensitive to oxidative stress at inflamed sites. Reduced PRL2 in phagocytes causes increased respiratory burst activity and enhances phagocyte bactericidal activity. PRL2 (Phosphatase Regenerating Liver 2) is highly expressed in resting immune cells, but is markedly downregulated by inflammation. *in vitro* experiments showed that PRL2 was sensitive to hydrogen peroxide (H_2_O_2_), a common damage signal at inflamed sites. In response to infection, PRL2 knockout (KO) phagocytes were hyper activated, produced more reactive oxygen species (ROS) and exhibited enhanced bactericidal activity. Mice with PRL2 deficiency in the myeloid cell compartment were resistant to lethal listeria infection and cleared the bacteria more rapidly and effectively. Moreover, *in vitro* experiments demonstrated that PRL2 binds to GTPase Rac and regulates ROS production. Rac GTPases were more active in PRL2 (KO) phagocytes than in wild type cells after bacterium infection. Our findings indicate that PRL2 senses ROS at inflamed sites and regulates ROS production in phagocytes. This positive feedback mechanism promotes bactericidal activity of phagocytes and may play an important role in innate anti-bacterial immunity.

## Introduction

Phagocytes are used by our immune system to remove and destroy pathogens (1, 2). To defend against pathogens, these specialized phagocytic immune cells use a process called “respiratory burst,” releasing reactive oxygen species (ROS) to degrade internalized microbes (3, 4). The key producer of ROS in phagocytes is the NADPH-oxidase complex which is made up of 5 phagocytic oxidase units (Nox2, p22phox, p40phox, p47phox, and p67phox) and a Rac GTPase (5). Under normal circumstances the NADPH complex is latent but it is activated to assemble during infection. In order to destroy microbes efficiently and avoid self-harm the activation of NADPH and the generation of ROS are precisely regulated (6). It has been shown that phagocytes in inflamed tissues produce ROS more quickly and generate more ROS than cells in normal tissues (7). However, the precise mechanisms by which ROS generation is controlled are still not fully elucidated.

The PRL (Phosphatase of Regenerating Liver) family of phosphatases are coded for by the protein tyrosine phosphatase IVA *(PTP4A)* gene and have 3 members, PRL1, PRL2, and PRL3 (8). PRLs have been validated as biomarkers and therapeutic targets in cancer and studies have shown that individual PRLs, especially PRL3, are expressed at high levels in variety of cancer cells and tissues (9, 10) and that overexpression of PRLs promote cell proliferation, migration, invasion, tumor growth and metastasis (11). Among the PRL family, PRL2 is the least studied member. While studies have reported that PRL2 is overexpressed in pancreatic, breast and lung cancer samples and its level is associated with tumor progression, there is still a lack of information available regarding the function of PRL2 (12). The expression of PRL2 is the most abundant of the three PRLs and PRL2 mRNA is almost ubiquitously expressed at high levels in normal adult human immune tissues, suggesting that PRL2 may have a specific role in immune function (13). Here we report that PRL2 is highly expressed in murine innate immune cells, acting as a ROS sensor and regulator. In innate phagocytes, PRL2 rapidly responds to the oxidative stress at the inflamed site by down regulating its own expression, leading to enhanced respiratory burst. This positive feedback mechanism promotes bactericidal activity of phagocytes and may play an important role in innate anti-bacterial immunity.

## Materials and methods

### Ethics statement

The conducts and procedures involving animal experiments were approved by the Animal Ethics Committee of Shanghai Jiao Tong University School of Medicine (project number 2012008, A-2016–028). It is according to the Regulations for the Administration of Affairs Concerning Experimental Animals (approved by the State Council of the People's Republic of China) and the Guide for the Care and Use of Laboratory Animals (Department of Laboratory Science, Shanghai Jiao Tong University School of Medicine, laboratory animal usage license number SYXK 2013–0050, certificated by Shanghai Committee of Science and Technology).

### Mice

Wide-type C57BL/6 mice were purchased from Shanghai Laboratory Animal Center, Chinese Academy of Sciences. Mice in which the *Ptp4a2* exon 4 are flanked by LoxP sites (*Ptp4a2*^*fl*/*fl*^mice, 129S_6_/S_v_E_v_ background) were generated by Shanghai Model Organism Center. C57BL/6 mice that carry a *Ptp4a2*^*fl*/*fl*^ gene were generated by backcrossing *Ptp4a2*^*fl*/*fl*^ mice to C57BL/6 mice for 10 generations. LysM^WT/cre^ B6 background transgenic mice were kindly provided by Feng Qian's Lab in School of Life Science, Fudan University. Ptp4a2^fl/fl^ B6 mice were crossed with LysM^WT/cre^ mice. Ptp4a2^fl/fl^LysM^Cre+^ mice (CKO) and their wild-type (WT) littermates (Ptp4a2^fl/fl^LysM^Cre−^ mice) are the offsprings of the Ptp4a2^fl/fl^ B6 mice and the LysM^WT/cre^ B6 mice. Mice were housed in the Shanghai Jiaotong University School of Medicine Animal Care Facilities under specific pathogen-free conditions.

### Protein extraction and immunoblotting

The thymus, bone marrow, spleen and lymph nodes were harvested from mice and passed through a cell strainer to generate single cell suspensions. The blood was collected from mice and the red blood cells were removed using ACK lysis buffer. Cells were used for protein extraction after being washed twice in phosphate-buffered saline (PBS). Whole cell lysates were prepared by resuspending cells in lysis buffer (150 mM NaCl, 10 mM Tris, pH 7.4, 0.1% SDS, 1% Triton X-100, 1% sodium deoxycholate, 100 μM Na_3_VO_4_, 5 mM EDTA, 1 mM PMSF) supplemented with 1x complete protease inhibitor cocktail (Roche), followed by determination of protein concentration by BCA assay (Pierce). Equal quantities of proteins were separated by SDS-PAGE, transferred to a nitrocellulose membrane, and blotted using specific antibodies [anti-PRL2 antibody (Millipore), anti-GAPDH antibody (Sigma), anti-Rac1/2/3, anti-β-actin, anti-iNOS and anti-Arginase1 antibodies (CST), anti-Myc antibody (Invitrogen)] and HPR conjugated anti-mouse or anti-rabbit IgG (CST). The membrane was developed using Pierce SuperSignal reagent (Pierce) and detected by ImageQuant LAS 4000 mini (GE).

### Primary neutrophil and macrophage isolation and generation

Morphologically mature neutrophils were purified from murine bone marrow by Percoll gradient centrifugation, as previously described (14). Briefly, bone marrow cells were harvested from mice using neutrophil isolation buffer (1 × HBSS without Ca^2+^ and Mg^2+^ containing 0.25% BSA). After RBC lysis, cells were layered on a 3-step Percoll gradient (81%, 62%, 55%), centrifuged at 1,200 g for 30 min at room temperature and the cells at the 81%:62% interface were collected and washed. To obtain neutrophils from an inflamed site, 2% (w/v) casein in PBS (2 mL for each) was injected i.p., in mice. Four hours later the peritoneal exudate was collected and neutrophils were isolated and purified as above. After purification, neutrophil viability was assessed as >95% by trypan blue staining. Purity was typically >80% as assessed by flow cytometry based on the forward and side scatter and high Gr1 staining.

Macrophages were isolated from the peritoneal cavity of naïve mice or from mice that had been injected with 2 ml of 4% thioglycollate i.p. for 72 h. Peritoneal cavity cells were collected using ice cold PBS, washed, suspended in Dulbecco's modified Eagle's medium (DMEM) containing 10% heat-inactivated fetal bovine serum (FBS), 2 mM L-glutamine, and 100 U/ml penicillin/streptomycin (D10), and plated in petri dishes. Non-adherent cells were removed 2 h later and the adherent macrophages were used for further experiment. The purity of macrophages was >95%, as determined by flow cytometry analysis using F4/80 and CD11b markers.

To generate bone marrow-derived macrophages (BMDMs), bone marrow cells were harvested from mice and cultured in D10 with 50 ng/ml M-CSF for 7 days. Macrophages were treated and collected using 5 mM EDTA in cold PBS, centrifuged and resuspended in D10, followed by seeding and resting for 24 h before functional assays. BMDMs were >95% CD11b^+^ and F4/80^+^ as determined by flow cytometry.

### Cell culture and transfection

The murine Raw 264.7 (ATCC), COS7 (ATCC) and HEK293T cell lines (ATCC) were cultured in DMEM containing 10% (vol/vol) heat-inactivated FBS, 2 mM L-glutamine, and 100 units/mL penicillin/streptomycin (D10). Transfections were performed using Attractene transfection reagent according to manufacturer's instruction (QIAGEN).

### Plasmid constructs

Mouse PRL2-pRK5 plasmid was generated by Dr. YH Chen' Lab (University of Pennsylvania). Full length PRL2 was generated from the cDNA clone by PCR and subcloned into pRK5 with Myc tag at the N-terminal. Myc-Rac1, Myc-Rac2, and HRasG12V plasmids were obtained from Dr. YH Chen's lab and were as described previously (15). All constructs and mutations were confirmed by DNA sequencing.

### *In vivo L. monocytogenes* infection model

Wild-type *Listeria monocytogenes* (10403s) were provided by Dr. H. Shen (University of Pennsylvania) and grown at 37°C in Brain-Heart-Infusion medium (Becton Dickinson). Mid-log-phase bacteria were used for the experiments. *Ptp4a2*^fl/fl^LysMCre^+^ mice and WT controls were infected i.v., with 3.75 × 10^5^ bacteria in 150 μl PBS. Mice were observed daily post-infection. For measurements of the bacterial burden, liver and spleens were homogenized 24 h after inoculation in 0.1% Triton in PBS, before plating serial dilutions of the homogenate on Brain-Heart-Infusion agar plates. The colonies were counted 24 h later. Serum alanine aminotransferase (ALT) was measured by Beckman-Coulter chemistry analyzer AU5800.

### Phagocytosis and killing assays

Phagocytosis and killing assays were performed in 12-well plates. A total of 1 × 10^6^ BMDMs were seeded in each well and cultured overnight in D10. Mid-log-phase *L. monocytogenes* or *E. coli* (DH5α) transformed with pRK5 plasmid, or fluorescently labeled 2 μm beads, were added to BMDMs at different multiplicity of infection (MOI). Centrifugation was performed at 500 g for 2 min to synchronize binding and internalization. For the phagocytosis assay following a 30 min incubation at 37°C the plates were rapidly washed with ice cold PBS twice. The cells were digested with 5 mM EDTA-PBS followed by 2% paraformaldehyde for fixing, and analyzed by flow cytometry. Cells incubated with bacteria were washed twice with PBS and lysed with 0.1% (v/v) Triton X-100 in PBS.

For the killing assay, 20 min post infection was considered to be the starting point of killing progress and the media was changed to fresh media containing 50 ng/mL gentamycin at this point. After 2 h the cells were washed and lysed with 0.1% (v/v) Triton X-100 in PBS. To determine the number of remaining intracellular bacteria, serial dilutions of the samples were plated on LB agar plates with ampicillin (*E. coli* transformed with PRK5 plasmid) or BHI agar plates with streptomycin (*L. monocytogenes*). The colonies were counted 24 h later.

### Detection of ROS

Neutrophil ROS production was measured by a luminol-dependent chemiluminescence assay. 3 × 10^5^ cells were plated in a 96-well luminometer plate (Coster) and prewarmed for 5 min. Pre-warmed fMLP (5 μM, Sigma-Aldrich) or Zymosan (100 μg/ml, Sigma-Aldrich) were added together with luminol (100 μM, Sigma-Aldrich) and HRP (20 U/ml, Sigma-Aldrich) and measurements started immediately. Chemiluminescence was measured at 2.5 min intervals for 30–60 min with a luminometer (BioTek Synergy HT microplate reader).

ROS production from BMDMs and Raw cells was detected using 2′,7′-dichlorofluorescein diacetate (DCFDA Sigma-Aldrich). Cells were incubated with the fluorogenic probe DCFDA for 30 min at 37°C in 5% CO_2_ and ROS was determined using a microplate reader.

### PAK pull-down

In order to measure Rac activity in mouse macrophages or transfected 293T cells, cells were washed in PBS and lysed in PBD lysis buffer (50 mM Tris pH 7.5, 10 mM MgCl2, 0.2 M NaCl, 0.5% NP-40, and 1x protease inhibitors cocktail) (Roche). The lysate was incubated with 20 μg of PAK-GST protein beads (Cytoskeleton) for 30 min at 4°C, washed and then subjected to Western blot.

### Immunofluorescence and confocal assay

COS7 cells were grown on chamber slides (Lab-Tec) for 24 h. Myc-PRL2 with Rac1-EGFP or Rac2-EGFP were co-transfected to COS7 cells. After 24 h of transfection, cells were washed twice with pre-heated PBS and fixed with 2% paraformaldehyde in PBS for 10 min at 37°C, followed by treatment with 0.1% saponin/0.3% BSA for 15 min, and blocking using 3% BSA for 45 min. The cells were then stained with anti-Myc Alexa 647 antibody (ebioscience) for 1 h, washed and mounted in ProLong Gold anti-fade reagent with DAPI (Molecular probes). Fluorescence was captured by a laser confocal microscope (Leica TCS SP8) at 63X magnification.

### Co-immunoprecipitation

To determine the interaction between PRL2 and Rac1/2, 293T cells were transfected with Myc-PRL2 or Myc-Rac1/Myc-Rac2, respectively. Cell lysates were prepared 24 h after transfection using lysis buffer (50 mM HEPEs, 150 mM NaCl, 1 mM EDTA, pH 7.0, 0.1% ICEPAL) supplemented with 1 × complete protease inhibitors mixture (Roche). Immunoprecipitation was performed using Dynabeads protein G (Invitrogen). In brief, 1.5 mg Protein G Dynabeads were coated with 5 μg anti-Myc antibody (Invitrogen) or Ig control for 1 h at room temperature with rotation. After removing unbound antibody, the bead-antibody complex was incubated with cell lysate overnight at 4°C with rotation. The captured Dynabead/Ab/Ag complex was washed 4 times with PBS and boiled in 2 × Laemmli buffer. The eluted proteins were subjected to 12% SDS-PAGE for Western blotting.

### Statistical analyses

Statistical differences in phagocytosis, bacterial killing and DCFH assay were analyzed by unpaired Student's *t-*test. Statistical differences in survival rate were analyzed by Log-rank (Mantel-Cox) test and area under curve (AUC) was analyzed using GraphPad Prism software.

## Results

### Innate immune cells from inflammatory sites show less PRL2 expression

**Figure 1:**
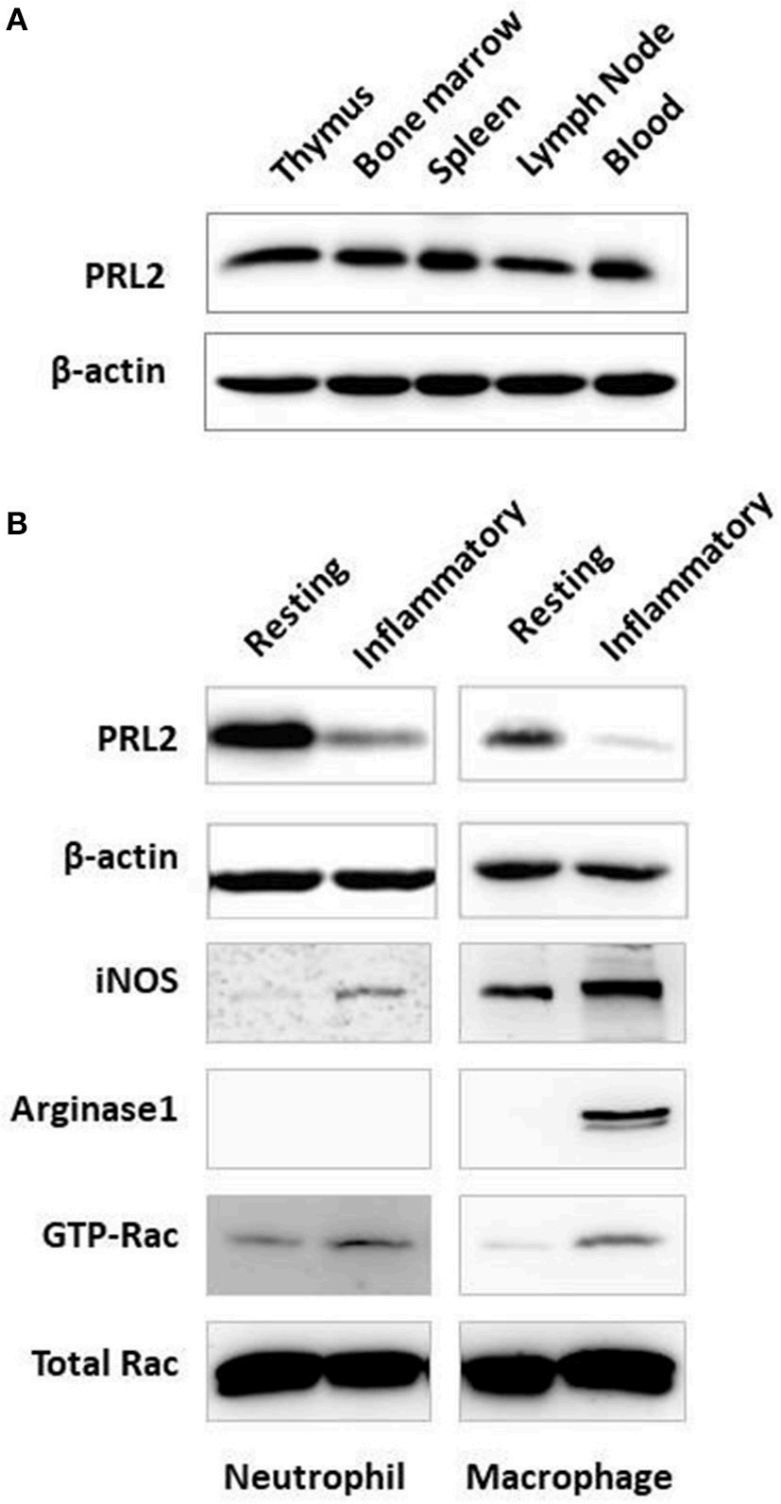
Expression of PRL2 in murine immune tissues and cells. **(A)** Lysates of mouse immune tissues were subjected to SDS-PAGE followed by immunoblot analysis using the indicated antibodies. **(B)** Neutrophils were purified from naïve mouse bone marrow (resting) or abdominal cavity exudate (inflammatory) as described in Material and Methods. Resting and inflammatory macrophages were collected from naïve mice or from the peritoneal cavity after thioglycollate-elicited peritonitis, respectively. Cell lysates were subjected to SDS-PAGE followed by immunoblot analysis using the indicated antibodies. Data are representative of two or three independent experiments.

To understand the functional role of PRL2 in the host immune system we analyzed the expression of PRL2 in cells from different immune tissues. Cells were prepared from central and peripheral immune tissues of normal mice and PRL2 expression levels were analyzed by western blot. Similarly to the expression profile in human tissues, PRL2 was widely expressed in mouse immune tissues and readily detected in thymus, bone marrow, spleen, lymph nodes and blood (Figure 1A).

We next analyzed PRL2 expression under normal and inflammatory conditions, using resting naïve cells or inflammatory peritoneal cells isolated after casein or thioglycollate (TG)-induced peritonitis. As shown in Figure 1B, neutrophils isolated from the inflammatory site showed significantly less PRL2 expression compared with resting neutrophils from naïve mouse bone marrow. A similar reduction in PRL2 levels was seen when analyzing thioglycollate—elicited peritoneal macrophages compared to naïve resident peritoneal macrophages (Figure 1B).

### PRL2 is susceptible to oxidative stress

**Figure 2:**
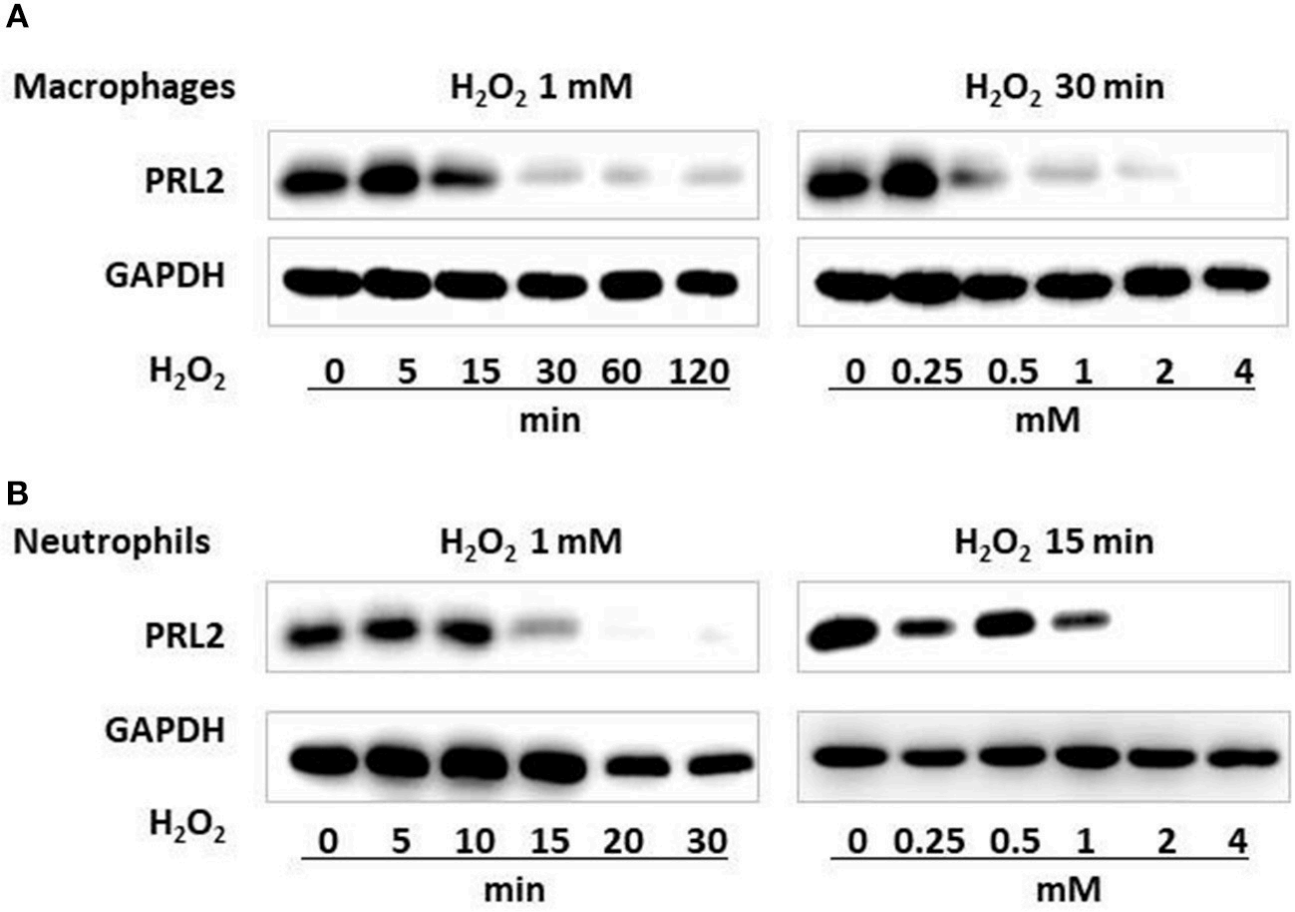
PRL2 is susceptible to oxidative stress. **(A)** Wild-type bone marrow-derived macrophages were treated with 1 mM H_2_O_2_ for the indicated times, or with the indicated concentrations of H_2_O_2_ for 30 min. Cell lysates were subjected to SDS-PAGE followed by immunoblot analysis using the indicated antibodies. **(B)** Mouse bone marrow neutrophils were treated with 1 mM H_2_O_2_ for the indicated times, or with the indicated concentrations of H_2_O_2_ for 15 min. Cell lysates were subjected to SDS-PAGE followed by immunoblot analysis using the indicated antibodies. Data are representative of three independent experiments.

Reactive oxygen species (ROS) are key signaling molecules in the process of inflammation (16) while PRL proteins belong to protein tyrosine phosphatases (PTPs) which are commonly susceptible to oxidative stress (17). To investigate if the reduced levels of PRL2 observed in inflammatory leukocytes was associated with oxidative stress, we examined PRL2 protein levels in murine bone marrow-derived macrophages (BMDMs) before and after treatment with hydrogen peroxide (H_2_O_2_). As shown in Figure 2A, upon treatment with H_2_O_2_ the protein levels of PRL2 were significantly reduced after 30 min, and the effects of H_2_O_2_ on primary BMDMs were dose and time-dependent. Similar effects were observed on primary neutrophils isolated from murine bone marrow where a significant decrease in the levels of PRL2 was observed after 15 min, with almost complete loss of PRL2 protein expression after 20 min (Figure 2B). The above results suggest that PRL2 responds to ROS rapidly and may be involved in immediate innate immune responses.

### PRL2 myeloid cell specific-deficient mice are resistant to lethal listeria infection

**Figure 3:**
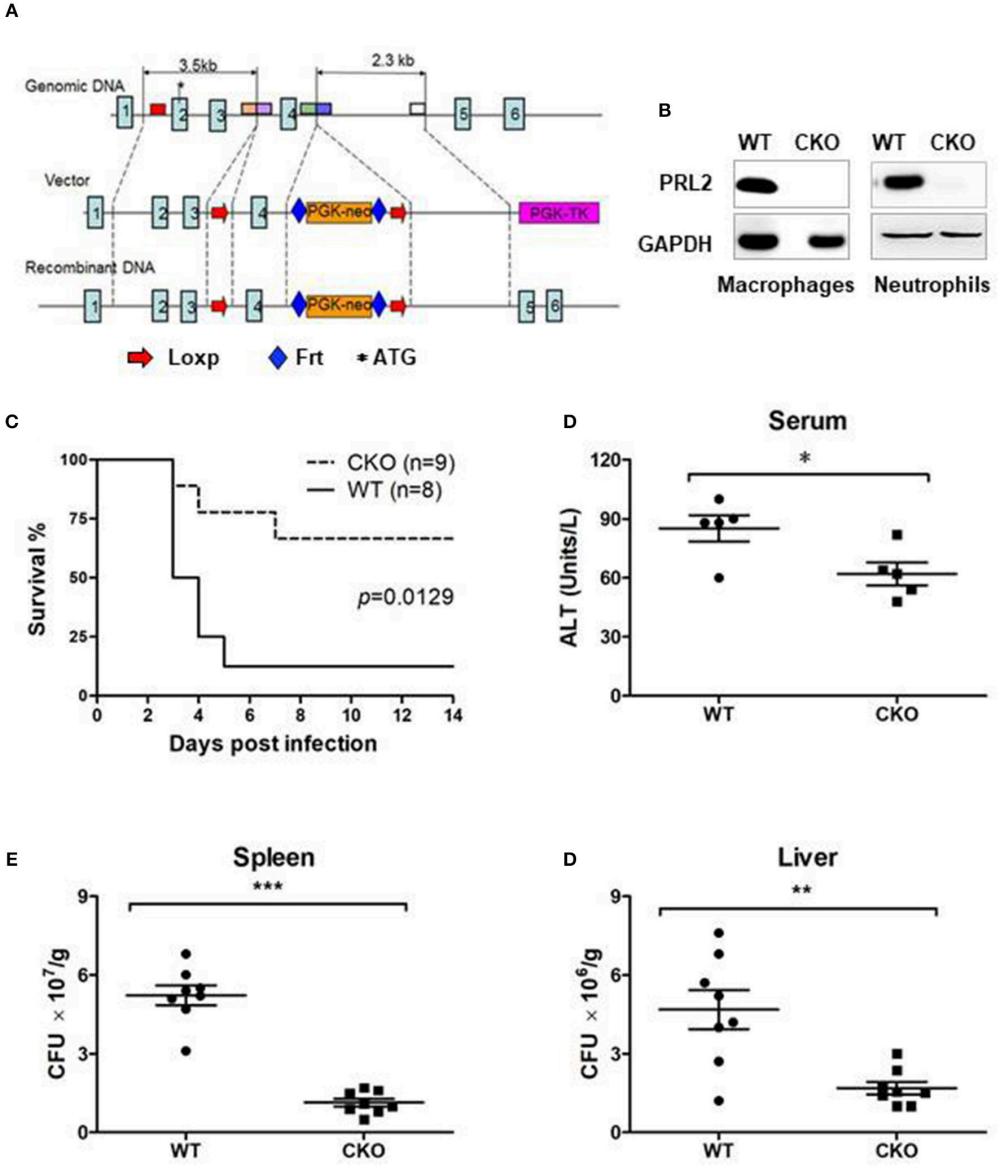
PRL2 myeloid cell-deficient mice are resistant to lethal listeria infection**. (A)** The strategy of Ptp4a2^fl/fl^ mice. LoxP sites were inserted at both ends of exon 4 of the *Ptp4a2* gene. **(B)** Peritoneal macrophages and bone marrow neutrophils were isolated from PRL2 myeloid cell conditional knockout mice (CKO) and their wild-type littermates (WT). Cell lysates were subjected to SDS-PAGE followed by immunoblot analysis using the indicated antibodies. **(C)** WT (*n* = 8) and PRL2 CKO mice (*n* = 9) were infected with 3.75 × 10^5^ CFUs of *L. monocytogenes* i.v and the survival monitored. **(D)** Serum ALT levels were measured 24 h post infection. **(E,F)** Liver and spleen CFUs were determined 24 h post infection by colony-forming unit assay. Data are pooled from 2 independent experiments. Error bars represent the SD. *P < 0.05, **P < 0.01, ***P < 0.005.

To further investigate the role of PRL2 in innate immunity, we generated PRL2 myeloid cell conditional knockout (CKO) mice. Targeting of *Ptp4a2* was achieved by introduction of LoxP sites flanking exon 4 of the *Ptp4a2* gene (Figure 3A). The resulting Ptp4a2^fl/fl^ B6 mice were crossed with LysM^WT/cre^ mice to generate Ptp4a2^fl/fl^LysM^Cre+^ mice where PRL2 is deleted in the myeloid-cell lineage (Figure 3B). The Ptp4a2^fl/fl^LysM^Cre+^ CKO mice were born and developed normally. Adult CKO mice had similar body size, and blood, spleen and bone marrow cellularity to that of their wild-type (WT) littermates (Ptp4a2^fl/fl^ LysM^Cre−^ mice) (Supplementary Figure 1). In order to analyse the myeloid cell development we generated bone marrow derived macrophages (BMDMs) from CKO and WT mice, and analyzed them by flow cytometry. No significant differences were observed between BMDMs with or without PRL2.

To investigate the role of PRL2 in the innate immune response we infected CKO and WT mice with a lethal dose of *L.monocytogenes*. The majority of WT mice succumbed within 5 days of infection, whereas the majority of the CKO mice survived (Figure 3C). The serum alanine transaminase (ALT) is an indicator of hepatic injury. After Listeria infection, the serum ALT levels were higher in WT than in CKO mice (Figure 3D). Mortality of WT mice was also associated with high titers of bacteria in liver and spleen while the listeria titers in the CKO mice was significantly lower at 24 h post infection (Figures 3E,F). Taken together, these results demonstrate that PRL2 act as an inhibitor of innate immunity to bacteria.

### PRL2 negatively regulates bactericidal activity of phagocytes

**Figure 4:**
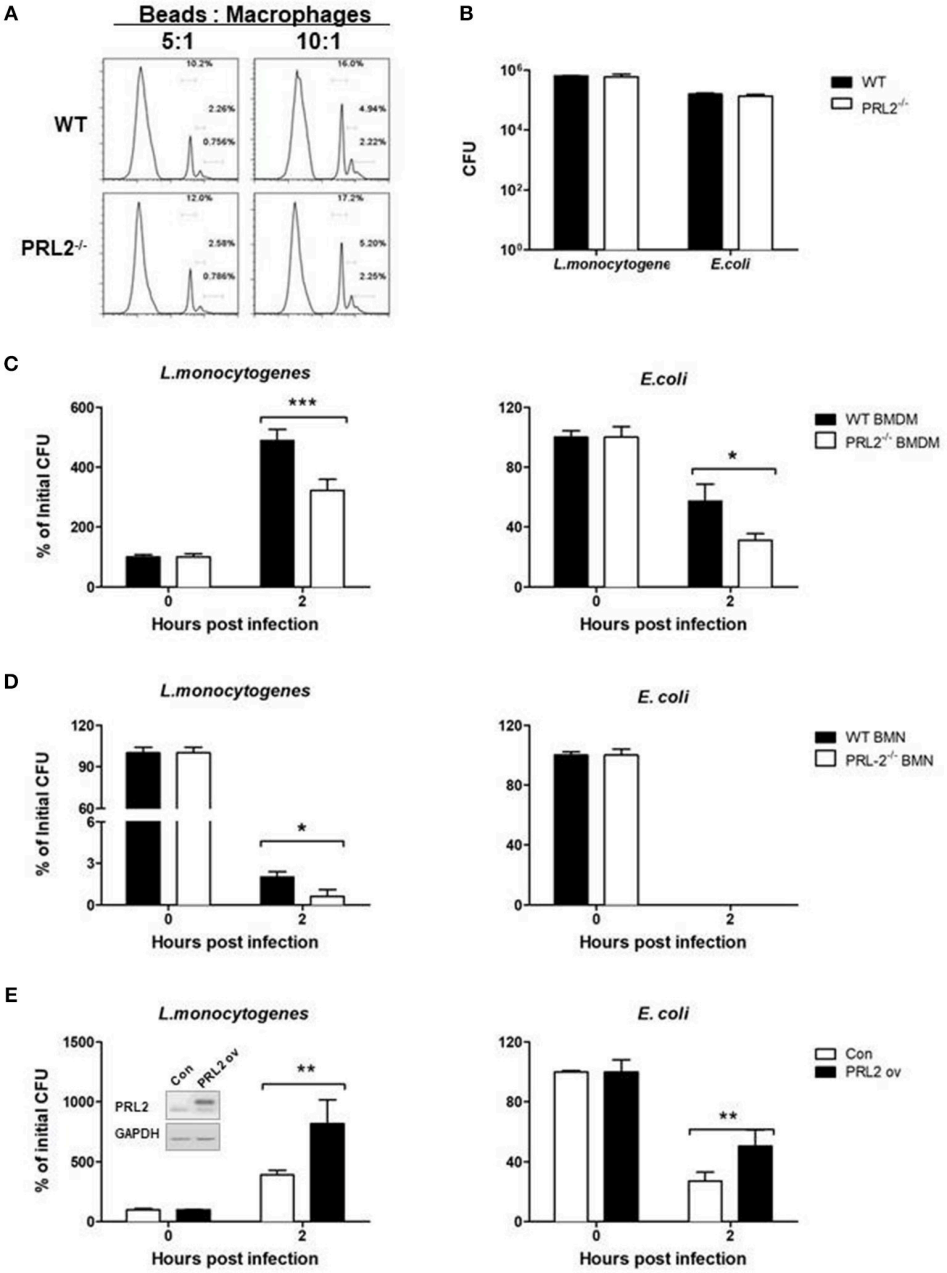
PRL2 negatively regulates bactericidal activity of phagocytes. **(A)** Bone marrow-derived macrophages (BMDMs) from PRL2 CKO mice and wild-type littermates were incubated with fluorescently labeled 2 μm beads at a ratio of 5:1 or 10:1 (Beads: cells) for 30 min, The phagocytosis function of BMDMs was evaluated by flow cytometer assay. **(B)** BMDMs from PRL2 CKO mice and wild-type littermates were incubated with *Listeria monocytogenes* (*L. monocytogenes*) or *Escherichia coli* (*E. coli*) at MOI 10 (bacteria:cell) for phagocytosis analysis. Surviving intracellular bacteria were determined by colony-forming unit assay. **(C,D)** BMDMs and bone marrow neutrophils (BMNs) from WT or CKO mice were infected with *L. monocytogenes* or *E. coli* at a ratio of 1:1 for 20 min. After washing cells were incubated for additional 2 h. Bacterial numbers in the cells were determined by colony-forming unit assay. **(E)** Raw 264.7 cells were transfected with PRL2, or a control plasmid, and incubated with *L. monocytogenes* or *E. coli* at a ratio of 1:1 for 20 min. After washing cells were incubated for additional 2 h. The intracellular bacteria were determined by colony-forming unit assay. Data are pooled from three to four independent experiments. *p < 0.05, **p < 0.01, ***p < 0.005.

Phagocytes play a major role in innate immune responses against bacteria through phagocytosis and killing of microbes. We analyzed the role of PRL2 in phagocytosis by incubating PRL2^−/−^ and WT macrophages with fluorescently labeled beads, followed by measurement of fluorescent-positive cells by flow cytometry. As can be seen in Figure 4A, there was no difference in phagocytic ability between PRL2^−/−^ cells and WT cells. Similarly, when the cells were incubated with *L.monocytogenes* or *E.coli* at a ratio of 1:10 for 30 min there was no difference in the number of bacteria taken up by the cells (Figure 4B). We next measured bactericidal activity of phagocytes. After 2 h of bacterial infection, PRL2-deficient macrophages killed *L. monocytogenes* and *E. coli* more efficiently than WT cells (Figure 4C). Killing of bacteria occurs very quickly in neutrophils, no *E.coli* survived in WT or PRL2 deficient neutrophils. As an intracellular bacterium, some *L. monocytogenes* can survive in WT and PRL2^−/−^ neutrophils. More *L. moncytogenes* survived in WT cells than in PRL2^−/−^ cells (Figure 4D). To further confirm the effect of PRL2 on bacterial killing, we overexpressed PRL2 in the murine RAW 264.7 macrophage cell line. Overexpression of PRL2 significantly increased bacterial survival in macrophages (Figure 4E). Taken together, the above results suggest that PRL2 negatively regulates bactericidal activity in phagocytes.

### PRL2 inhibits oxidative burst in bacterial infection

**Figure 5:**
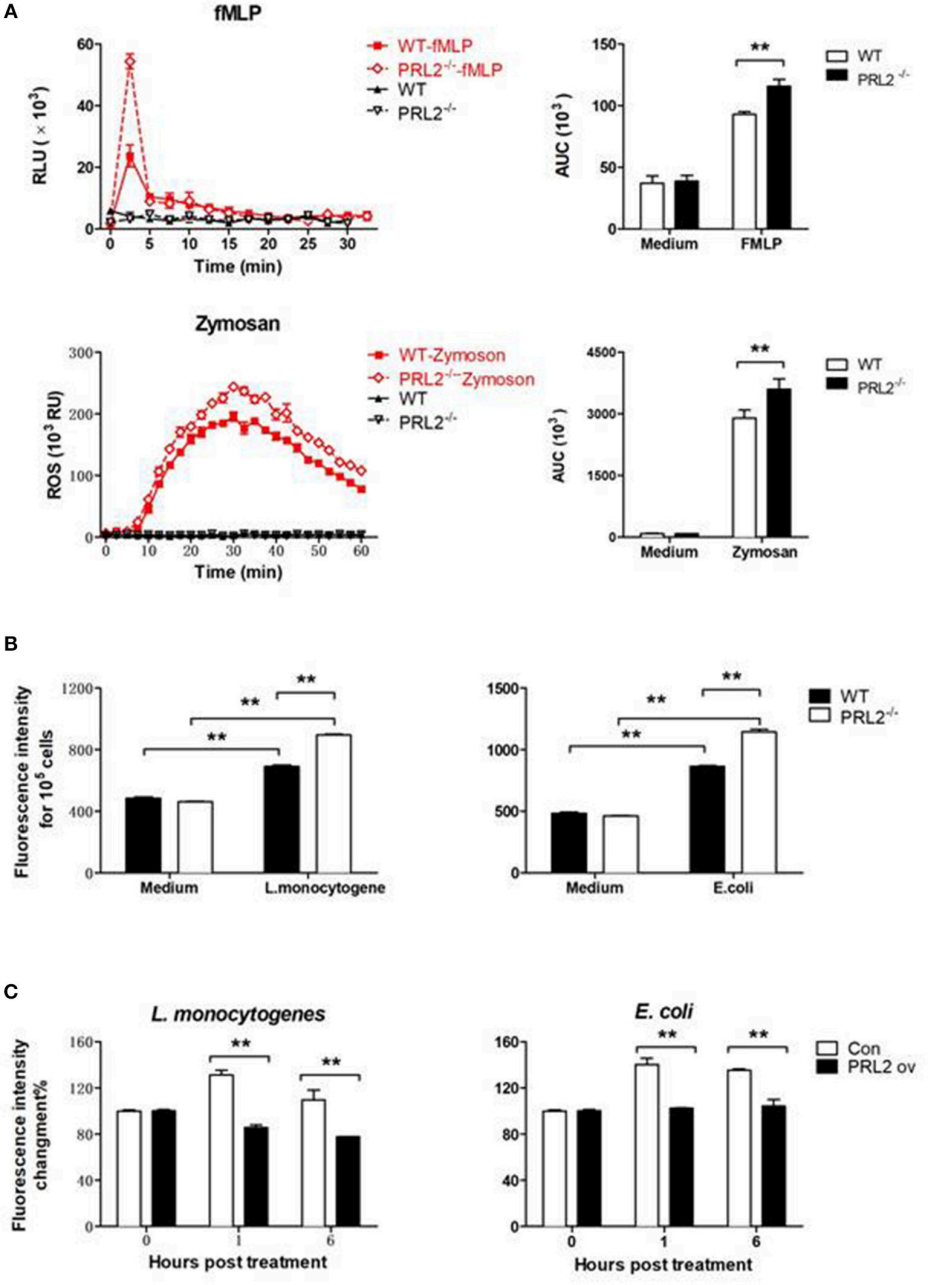
PRL2 controls oxidative burst. **(A)** Neutrophils collected from PRL2 CKO mice and wild-type littermates were stimulated with 5 μM fMlP or 100 μg/ml Zymosan and ROS production was measured by chemiluminiscence assay as described in section Materials and Methods. Left, ROS kinetic plots of a representative experiment. Right, averaged area under cure (AUC) from 3 independent experiments. **(B)** WT and PRL2^−/−^ BMDMs were treated with bacteria as indicated in graph for 30 min and ROS production was measured by fluorescent staining. **(C)** Raw264.7 cells were transfected with PRK5 or Myc-PRL2 PRK5 for 24 h. Cells were treated with *L. monocytogenes* or *E. coli* at MOI = 10 (bacteria vs. cells) for 30 min. ROS production was measured by fluorescent staining assay as above. Results shown are means ± SEM and are representative of more than three independent experiments. **p < 0.01.

The bacterial killing ability of phagocytes is highly related to oxidative burst capacity. In order to evaluate the role of PRL2 on oxidative burst we stimulated cells with pathogen components and measured ROS production using a horseradish peroxidase (HRP) enhanced chemiluminescence (CL) system. PRL2^−/−^ and WT neutrophils were stimulated with the bacterial peptide N-formyl-methionyl-leucyl-phenylalanine (fMLP) or Zymosan A from *Saccharomyces cerevisiae*, two well-known stimuli of NADPH-oxidase activation. In response to fMLP stimulation, neutrophils quickly increased ROS generation. The peak values of chemiluminescence were observed within 5 min of stimulation, with PRL2^−/−^ neutrophils producing significantly more ROS than WT cells. When neutrophils were stimulated with Zymosan the peak values appeared around 30 min after stimulation and PRL2^−/−^ neutrophils again generated more ROS than WT cells (Figure 5A).

ROS production in macrophages was detected by incubating cells with H_2_-DCFDA and measuring the intracellular levels of ROS by using a fluorescence microplate reader. As shown in Figure 5B, incubating macrophages with either *L. monocytogenes* or *E. coli* induced a strong ROS response, and PRL2^−/−^ macrophages producing significantly more ROS than WT cells. On the other hand, ectopic overexpression of PRL2 significantly inhibited ROS production in macrophages (Figure 5C).

In phagocytes, oxygen-dependent killing can be mediated by ROS or nitric oxide (NO). NO is produced by inducible NO synthase (iNOS) which require the cofactor NADPH (18). We measured iNOS expression and NO production in WT and PRL2 deficient macrophages. In response to bacterial component stimulation, PRL2 deficient macrophages produced more NO than WT cells, while the expression of iNOS was similar (Supplementary Figure 2). The above results suggested the activity of NADPH might be important in PRL2 associated bacterial killing.

### PRL2 binds to rac GTPase and regulates its activation

**Figure 6:**
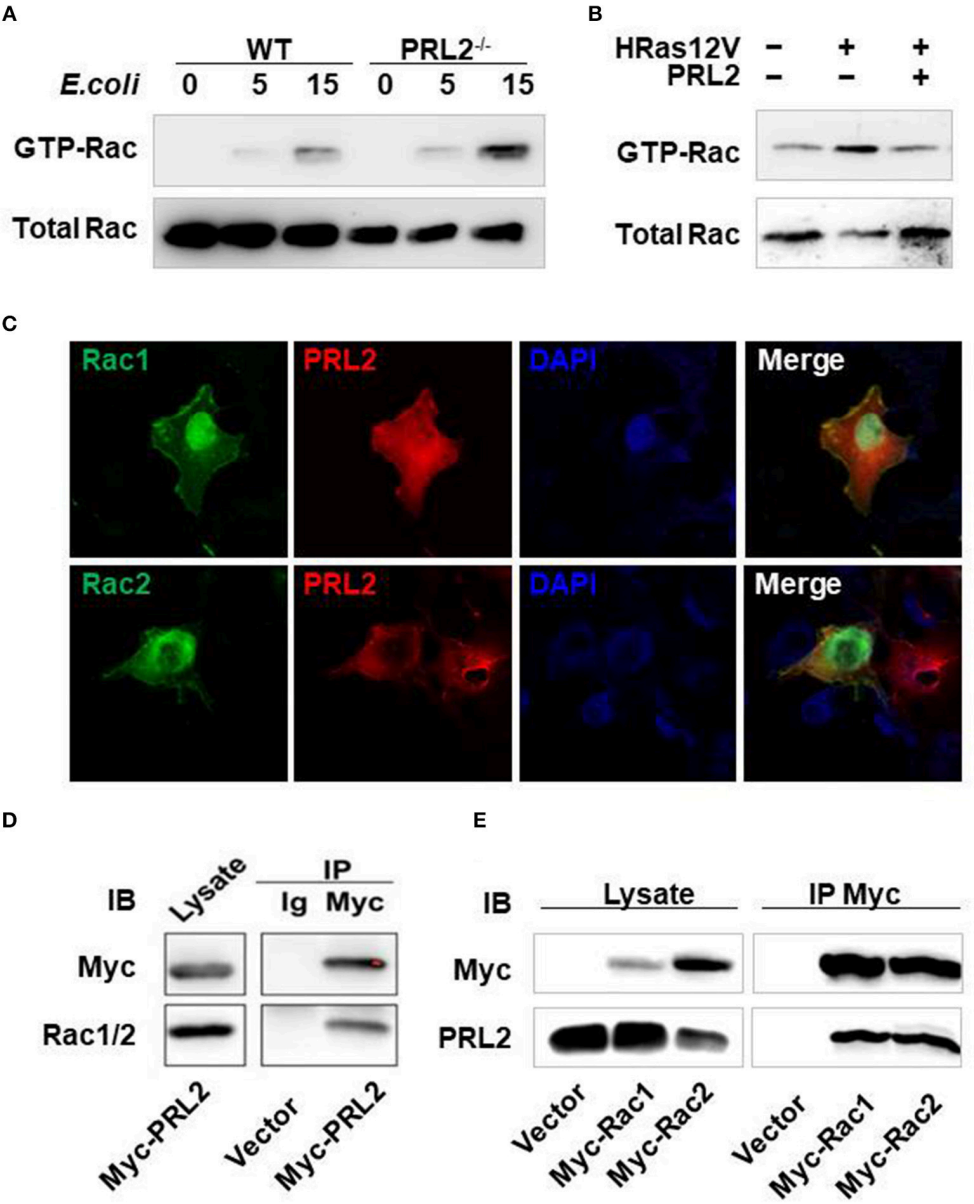
PRL2 binds to, and regulates the activity of, Rac GTPase. **(A)** WT or PRL2^−/−^ BMDMs were treated with *E.coli* (MOI = 10, bacteria vs. cell) for the indicated times. Cell lysates were subjected to pull-down using PAK GST beads. Lysate from pull-down and total lysate were subjected to SDS-PAGE followed by immunoblot assay with anti-Rac antibody. The experiments were repeated 2 times with similar results. **(B)** 293T cell was transfected with HRas12V or HRas12V and PRL2 for 8 hours. Active and total Rac was detected as above. The experiments were repeated 2 times with similar results. **(C)** COS7 cells on chamber slides were co-transfected with Rac1-EGFP, Rac2-EGFP and Myc-PRL2 and stained with anti-Myc Alexa 647 antibody (red). Images are representative of two independent experiments. **(D)** Lysate of 293T cells transiently transfected with Myc–PRL2 pRK5 constructs were immunoprecipitated with anti-Myc beads or anti-IgG beads. Immunoprecipitates and total cell lysate were subjected to SDS-PAGE for western blot. **(E)** 293T cells were transfected with pRK5 vector or expression plasmids for Myc-tagged Rac1 or Rac2. Cell lysates were collected for co-immunoprecipitation with anti-Myc beads 24 h post transfection. Immunoprecipitates and total cell lysates were treated as above. The experiments in panel d and e were repeated at least three times with similar results.

In antibacterial immune responses ROS are mainly generated by the NADPH-oxidase complex which is made up of 5 phagocytic oxidase units and a Rac GTPase. It has been reported that Rac GTPase is involved in the PRL signaling pathway (11), so we asked whether PRL2 could regulate Rac activation in innate immune responses as well. To address this question, we took 2 complementary approaches. First, we tested the effect of PRL2 deficiency on Rac activation in macrophages using a PAK pulldown assay. When cells were stimulated with *E. coli*, activated Rac GTPase was strongly induced in PRL2^−/−^ macrophages (Figure 6A). Second, we tested the effect of PRL2 in 293T cells that did, or did not, express HRas12V. Rac serves as an essential downstream component of the signaling pathway by which oncogenic RAS induces cell transformation (19) and HRas12V is a mutation which replaces the amino acid glycine with the amino acid valine at position 12. This altered HRAS protein is permanently active within the cell and causes Rac activation (15). As shown in Figure 6B, we found that PRL2 transfection inhibited HRas-induced Rac activation.

There are two members of Rac GTPases, Rac1 and Rac2. To further investigate the relationship between PRL2 and Rac we co-overexpressed Rac1/Rac2 and PRL2 in COS7 cells and measured their subcellular localization using immunofluorescence. Both Rac and PRL2 are membrane proteins. As expected, Rac1/Rac2 was enriched at the plasma membrane where it co-localized with PRL2. Co-localization of PRL2 and Rac was also observed in the cytosol. Overexpressed Rac1/Rac2 was also enriched in nucleus while PRL2 was distributed mainly at the nuclear envelope (Figure 6C). Since PRL2 and Rac were co-localized both in the cell membrane and the cytosol, we tested whether PRL2 could bind to Rac using a co-immunoprecipitation (Co-IP) assay. First, myc-PRL2 was expressed in 293T cells and upon blotting using an anti-Rac1/2 antibody a strong PRL2 signal was detected in the precipitates (Figure 6D), indicating that PRL2 interacted with endogenous Rac protein. Second, we expressed Myc-tagged Rac1 or Rac2 in 293T cells and detected endogenous PRL2 protein. We found that PRL2 interacted with both Rac1 and Rac2 (Figure 6E).

## Discussion

PRL proteins represent a group of protein tyrosine phosphatases that has been implicated in the development and metastasis of various types of cancer, however, little is known about their function in immune system. Here we report that PRL2 plays an important role in the innate immune response by sensing ROS and regulating ROS production. PRL2 is highly expressed across all tissues and organ systems (13). Orthology data show that PRL2 is highly conserved in mammalians and contain homologs in protozoa, worms, insects and vertebrates suggesting it may have a critical function. ROS represent an evolutionary ancient part of the innate immune response for fighting invading microbes (20). They are a highly reactive group of oxygen-containing molecules which act as important signaling messengers to regulate various biological and physiological processes, including certain immune response mechanisms (7). In this study, we revealed a relationship between mammalian PRL2 and ROS. We propose that PRL2 is a controller of respiratory burst. High levels of PRLs in resting leukocytes maintain redox homeostasis under normal conditions, while under inflammatory conditions reduced levels of PRL2 promotes oxidative burst in order to damage invading pathogens.

Data from PRL2 genomic knockout (KO)mice has shown that deletion of PRL2 leads to retarded growth both at birth and adult stage. PRL2 genomic KO mice are 20% smaller compared to their wild-type littermates throughout their adulthood, although their bone marrow cellularity is normal when normalized to total body weight (21). PRL2 was found to be a repressor of PTEN and required for a number of development processes (placenta formation, spermatogenesis and stem cell self-renewal) (21–23). In this study, we have focused on the role of PRL2 in innate immunity. We generated PRL2 myeloid cell-specific conditional knockout mice (CKO) and found that they display normal body weight as well as normal blood, spleen and bone marrow cellularity. Both *in vitro* data, using cells, and *in vivo* data, using *L.monocytogenes* infection, suggest PRL2 is involved in the innate immune response rather than in innate immune cell development. At the molecular level, we found the colocation of PRL2 and Rac on cell membrane and in cytosol. Co-IP data suggested PRL2 bind with Rac1 and Rac2. The most important source of ROS in phagocytes is NADPH-oxidase which is made up of 5 phagocytic oxidase units and a Rac GTPase (24). In the resting state, the oxidase units and Rac are separated; upon cell stimulation they assemble to form the active enzyme. This may explain our results where PRL2-deficient phagocytes only show differences after stimulation and not in a resting state.

PRLs share the CX5R active site, P-loop, and WDP loop motifs typical of PTPs, while the presence a CAAX prenylation motif next to a polybasic region make them a unique subfamily of PTPs (25). PTPs commonly possess a unique cysteine (cys) residue that is highly sensitive to oxidation by ROS (17) and it has been reported that the oxidation of PRL1 and 3 induces both intramolecular and intermolecular disulfide bond formation and that the biological function of PRLs can be regulated by oxidation (26). It is well established that ROS contributes to both physiological and pathological conditions via its involvement in redox signaling and oxidative stress (7). Here, we focus on the role of PRL2 in inflammation. Neutrophils and macrophages from inflamed sites expressed less PRL2 protein than cells from normal tissues. We found that the reduced levels of PRL2 were associated with high ROS levels in the tissue environment. In response to H_2_O_2_ treatment the PRL2 protein expression levels in neutrophils and macrophages were diminished within 15~30 min suggesting that the stability of the PRL2 protein may be altered under conditions of oxidative stress. It has been reported that the cys residue at the active site and CAAX terminal of PRL are both sensitive to oxidation and that the complete oxidation of full-length PRL leads to protein precipitation (27, 28). This may contribute to the instability of PRL2 under oxidative stress.

ROS are used by the immune system as weapons against pathogens; however ROS may also cause tissue damage. Precisely regulating the intensity and timing of ROS production is critical for the host antibacterial immune response. Our findings may reveal a basic ROS regulation signal in animals and the identification of PRL2 functions in innate immunity may be useful in providing novel insights into the mechanisms of ROS generation and regulation, and might eventually lead to the development of more effective therapies against infectious diseases or for the control of immunopathogenic responses.

## Author contributions

CY and ZW wrote the paper. ZW, CY ,and CW designed the experiments. YF, CW, XD, JW, LT, and JP performed and analyzed the data. WZ, YW, XG, and GC contributed reagents. GC and XG read the paper, and ZW oversaw the project.

### Conflict of interest statement

The authors declare that the research was conducted in the absence of any commercial or financial relationships that could be construed as a potential conflict of interest.
