# Supplementary material for: PRL2 Controls Phagocyte Bactericidal Activity by Sensing and Regulating ROS
Source: Front Immunol. 2018 Nov 13;9:2609. doi: 10.3389/fimmu.2018.02609 (PMC6244668; doi:10.3389/fimmu.2018.02609)
Supplement: Supplementary file 1 [file Data_Sheet_1.PDF]

Supplementary Data

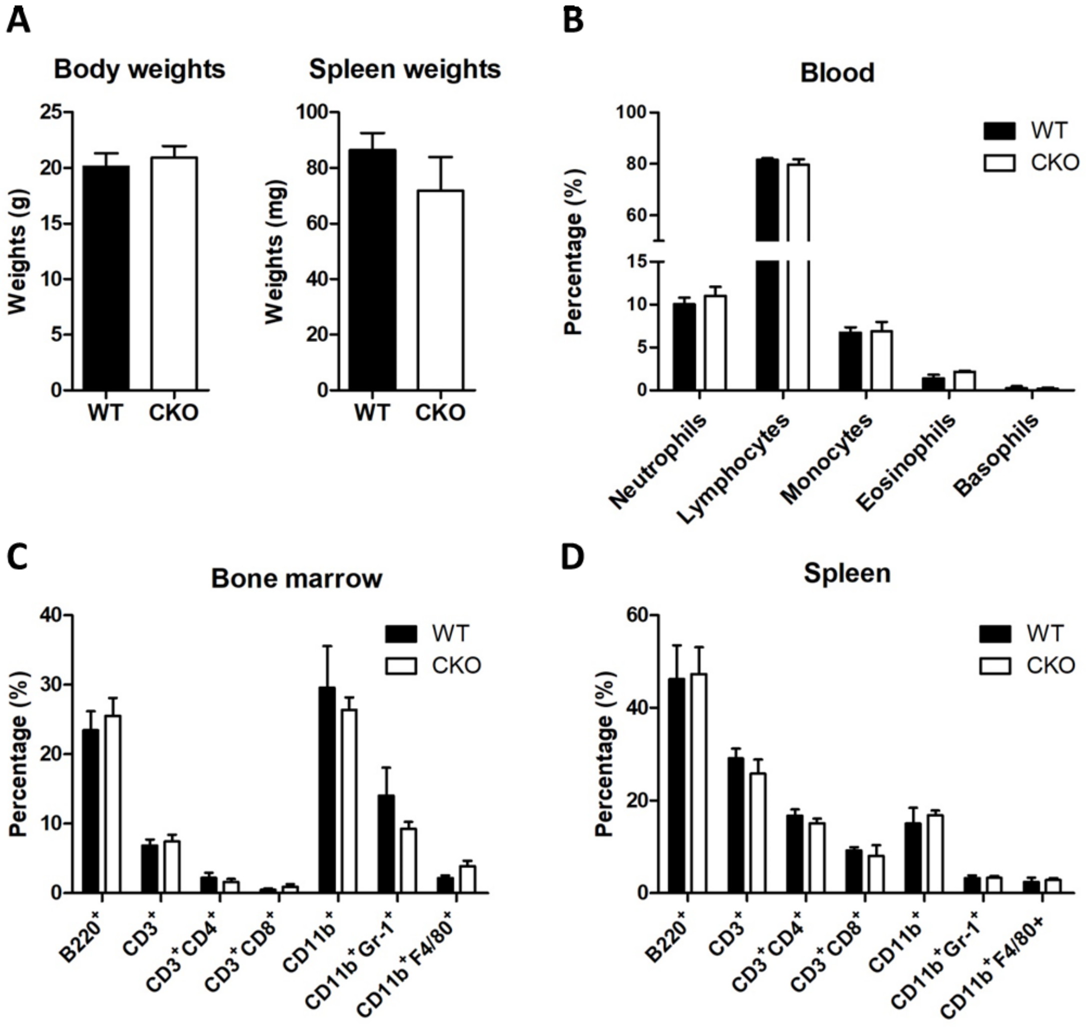

Supplementary figure 1. Analysis of body weights, spleen weights and immune cell subset in blood, bone marrow and spleen of naïve WT and PRL2 CKO mice.

(A) Body weights and spleen weights of naïve WT and PRL2 CKO mice (n=8). **(B-D)** Single-cell suspension of white blood cells, bone marrow and spleen were stained with the indicated surface marker and analysed by FACS. Percentages of cells in the total population are shown. WT, n=5; KO, n=5. All the values are mean±SEM.

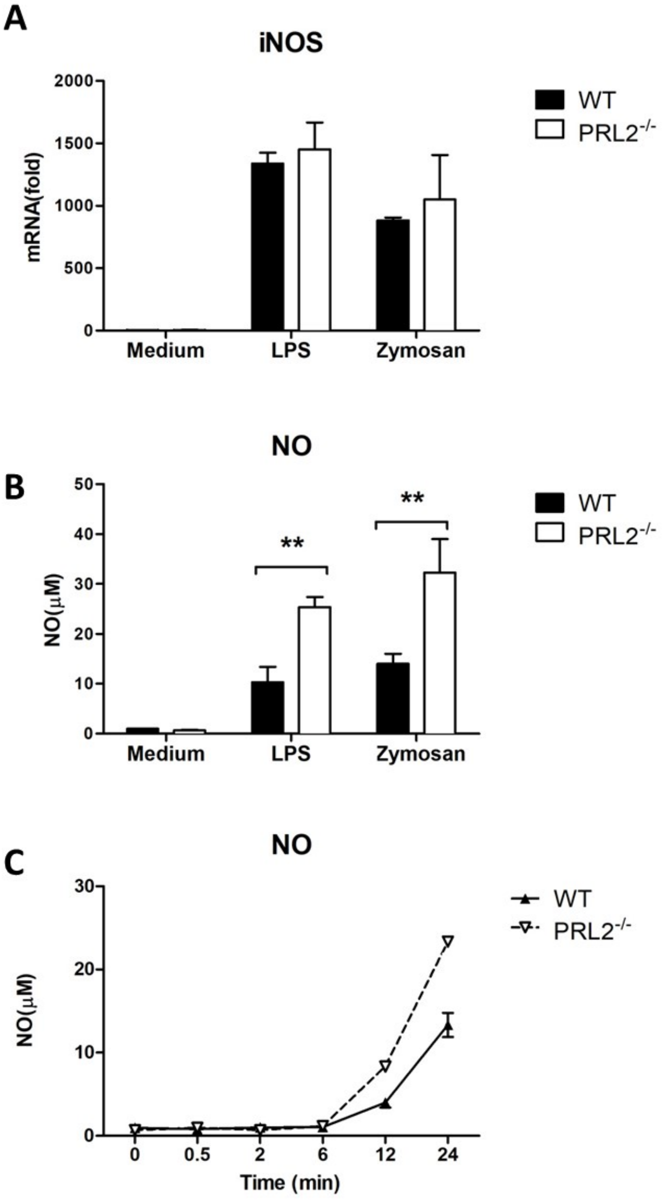

Supplementary figure 2. iNOS expression and NO production in WT andPRL2 KO macrophages. **(A)** WT and PRL2-/- BMDMs were stimulated with LPS (100 ng/ml) or Zymosan (100μg/ml) for 2 h. Total RNA was extracted and iNOS mRNA levels were determined by Real time PCR. iNOS expression levels were normalized to endogenous control. Results shown are means ± SEM and are representative of three independent experiments. **(B)** WT and PRL2-/- BMDMs were stimulated with LPS (100 ng/ml) or Zymosan (100 μg/ml) for 24h. Culture supernatants were analyzed for NO levels using Griess reaction. Results shown are means ± SEM and are representative of three independent experiments. ** $p<0.01$ **(C)** WT and PRL2-/- BMDMs were stimulated with LPS (100 ng/ml) for the indicated periods of time. NO levels in culture supernatants were measured using using Griess reaction.
